# Retraction Note: Long non-coding RNA TPT1-AS1 promotes cell growth and metastasis in cervical cancer via acting AS a sponge for miR-324-5p

**DOI:** 10.1186/s13046-022-02418-x

**Published:** 2022-06-22

**Authors:** Hui Jiang, Guanqun Huang, Nianzhang Zhao, Ting Zhang, Mengni Jiang, Yueming He, Xinke Zhou, Xianhan Jiang

**Affiliations:** 1 Department of Abdominal Oncology, The Fifth Affiliated Hospital of Guangzhou Medical University, Guangzhou, 510700 China; 2 Department of Gynaecology, The Fifth Affiliated Hospital of Guangzhou Medical University, Guangzhou, 510700 China; 3 Department of General Surgery, The Fifth Affiliated Hospital of Guangzhou Medical University, Guangzhou, 510700 China; 4 Department of Anesthesia, The Fifth Affiliated Hospital of Guangzhou Medical University, Guangzhou, 510700 China

**Retraction Note: J Exp Clin Cancer Res 37, 169 (2018)**

**https://doi.org/10.1186/s13046-018-0846-8**

The Editor-in-Chief has retracted this article at the corresponding author's request. After publication, concerns were raised regarding image overlap between the figures in this article and previous publications from different groups, specifically:

- Fig. 2d appears to overlap with Fig. 8b in [1]
- Fig. 3e appears to overlap with Fig. 4c in [2]

Additionally, in Fig. S1, the mean tumour weights in the TPT1-AS1 and sh-Vector groups appear to exceed the recommended tumour weight limit of 10% of initial bodyweight. No statement on Ethics approval for the use of animals is present in the article.

The authors have been unable to retrieve the original data or ethics documents to address these concerns.

Guanqun Huang, Nianzhang Zhao, Ting Zhang, Mengni Jiang, Yueming He and Xinke Zhou agree to this retraction. Hui Jiang and Xianhan Jiang have not responded to any correspondence from the publisher about this retraction.

## References

[1] Xu Q, Liu X, Liu Z (2017). MicroRNA-1296 inhibits metastasis and epithelial-mesenchymal transition of hepatocellular carcinoma by targeting SRPK1-mediated PI3K/AKT pathway. Mol Cancer.

[2] Wang Z, He S, Guo P, Guo X, Zheng J (2017). MicroRNA-1297 inhibits metastasis and epithelial-mesenchymal transition by targeting AEG-1 in cervical cancer. Oncol Rep.

